# Reducing zoonotic avian influenza transmission at household poultry slaughter using a behaviour change tool for limited literacy audiences

**DOI:** 10.1111/zph.12993

**Published:** 2022-09-06

**Authors:** Andrew A. Clark, Samah Eid, Mohamed K. Hassan, Kip Carter, David E. Swayne

**Affiliations:** ^1^ International Veterinary Consultant Pendleton Oregon USA; ^2^ Reference Laboratory for Quality Control of Poultry Production Animal Health Research Institute Giza Egypt; ^3^ Educational Resources, College of Veterinary Medicine University of Georgia Athens Georgia USA; ^4^ Southeast Poultry Research Laboratory, U.S. Department of Agriculture U.S. National Poultry Research Center, Agricultural Research Service Athens Georgia USA

**Keywords:** avian influenza, behaviour change, limited literacy, one health, safe poultry slaughter, zoonosis

## Abstract

Human infections in Egypt with highly pathogenic avian influenza (HPAI) likely due to airborne transmission of HPAI virus (HPAIV) during home slaughter of poultry predominately affect women and children, who are the primary caregivers of household poultry. This study developed a safe contained poultry slaughter procedure to reduce airborne HPAIV and zoonotic infections and simultaneously created an educational outreach tool for teaching the modified procedure. The tool designed for limited literacy audiences used two illustrated posters and handouts for teaching the safe contained poultry slaughter procedure. The posters were developed with advice of animal health professionals and then refined by target audience women's focus groups. These women's focus groups proved to be the critical step for assuring the understanding, acceptance, effectiveness and accuracy of the outreach tool. The safe contained poultry slaughter procedure was designed to be low or no cost, sustainable by using a universal implement found in village households and designed as a minor variation of standard poultry halal slaughter. It was crafted to be culturally appropriate and religiously acceptable.

**Impacts**

- A practical, halal‐compliant, poultry slaughter procedure was developed to reduce airborne virus‐laden particles responsible for human high pathogenicity avian influenza virus inhalation exposure
- A behavioural change outreach tool using colourful illustrated panels showing the steps of the safe contained poultry slaughter procedure was created for use on posters and handouts in limited literacy educational programs
- Veterinary health experts and target user group inputs provided important inputs in the development of the outreach tool design. In addition, end user critique by village women's focus groups provided final practical recommendations that resulted in an effective limited literacy educational program.

## 1 INTRODUCTION

The appearance of H5N1 Gs/GD lineage of highly pathogenic avian influenza (HPAI) in 1996 in southern China began the global arrival of human zoonotic infection with pandemic potential (Xu et al., 1999). Between 1997 and 2020, this lineage of H5 HPAIV was responsible for 864 human cases including 456 fatalities (WHO, 2022). Most of the cases occurred in low‐income countries in Asia and Africa and were associated with poultry contact in Live Poultry Markets (LPM) or at home (Cox et al., 2017; WHO, 2022).

The H5N1 HPAI cases have created a socioeconomic crisis in Egypt where most cases involved women and children, the primary caregivers and processers of household poultry for family consumption. Infections have appeared in both poultry and humans since 2006 with a peak of 136 human cases in 2015 (Castellan, 2006; WHO, 2022). The H5N1 HPAI epizootic has killed or resulted in culling of millions of poultry in Egypt, and although men handled and disposing of the infected poultry and their carcasses during stamping‐out programs, 39% of cases and 83% of deaths were in women 15 years and older (Kandeel et al., 2010). At the village level, household poultry husbandry and processing are primarily female activities with associated close poultry contact to children (FAO, 2016a, 2016b). Household poultry contribute to family nutrition and income and are often the principal source of independent income for women, thereby of great socioeconomic importance (Abdelwhab & Hafez, 2011; Fasina et al., 2010).

The H5N1 HPAIV has been recovered from air samples taken at LPMs of Hong Kong suggesting inhalation as a possible route of human exposure (Zhou et al., 2016). Furthermore, experimental studies in a high‐biocontainment laboratory using ferrets as the animal model of human transmission and infection demonstrated that the slaughter of H5N1 HPAIV‐infected chickens generated airborne virus and exposure of ferrets to the same air space resulted in virus transmission and infection (Bertran et al., 2017). Performance of the first two steps (kill and scald) of the chicken slaughter process in a containment vessel such as a plastic bag, covered‐pot or covered‐bucket, or use of influenza vaccinated chickens in the study prevented or reduced airborne virus and prevented HPAIV transmission and infections in ferrets exposed to the same air space (Bertran et al., 2018). These data suggest human infections resulted from inhalation exposure to airborne virus‐laden particles of dust, manure or blood generated during the slaughter process of HPAIV‐infected chickens.

This study had the goal to translate the research findings into a modified, practical home poultry slaughter procedure that would reduce airborne HPAIV, and simultaneously create an educational outreach tool to communicate the modified slaughter procedure. The desired outcome was behaviour change in the at‐risk population of women and children that would reduce potential exposure to HPAIV and produce a collective reduction in human HPAIV infections.

## 2 MATERIALS AND METHODS

### 2.1 Safe contained poultry slaughter procedure

Traditional household halal slaughter has five basic steps: (1) kill; (2) scald to loosen feathers; (3) manual defeathering; (4) evisceration and butchering; and (5) clean‐up. In village households, halal slaughter of poultry is typically performed outdoors by severing the carotid arteries and jugular veins followed by involuntary postmortem muscle contractions on the dry ground near the dwelling. Because HPAIV infection status is unknown, slaughter of infected but asymptomatic household birds can generate a plume of virus‐laden aerosols and droplets containing a mixture of blood, saliva, faeces, and/or feather dander along with or attached to dust particles, creating conditions for potential inhalation, transmission and zoonotic HPAIV infection.

The target group for behaviour change was low‐income people with limited reading ability and strong traditions deeply rooted in culture and religion. Factors considered in the modified slaughter procedure included technical feasibility, effectiveness in prior experimental studies, suitability for use at village level, acceptability within local cultural and religious community, affordability in the low‐income setting, sustainability in the long term and maximal use of locally sourced equipment. Fieldwork was oriented towards discovering acceptable options for capture of airborne virus generated during slaughter while concurrently exploring outreach tool design. These intertwined phases were to first develop the safe contained poultry slaughter procedure and second to develop usable, effective, affordable behaviour change educational materials utilizing visual illustrations with minimal reliance on written instructions and reading. Effective communication with limited literacy audiences was the critical consideration for adoption and use of the safe contained poultry slaughter procedure.

For effective implementation of proposed modifications, a critical component of the work was to seek advice from the at‐risk target audience about modification of the slaughter process and development of the communication tool. Therefore, women's focus groups were formed, composed of adult women who own household poultry flocks and who perform manual slaughter themselves. Their first‐hand experience was considered an asset. This approach of consultation with these groups and seeking their advice proved beneficial for creation of acceptable modifications to the slaughter procedure, and also for composition of accurate, informative behaviour change outreach materials.

The Reference Laboratory for Quality Control of Poultry Production (RLQP) in Giza was operations base for planning, implementation and collection of field trial data. Five field trials were designed to develop and test the modified slaughter methods and collect information for outreach tool composition. All field trials were performed with HPAIV‐negative poultry.

Prior to any field trials, meetings were held at RLQP to clarify objectives and the work plan to be accomplished with each trial. Initially, data from the field trials were verbal from participants to the research team. First, there were explanations by the research team, then informal back and forth conversation, questions, answers, clarifications and specifics as the experimentation progressed. In each activity, there was active discussion between researchers and villagers for understanding and practicality of specific steps in the safe contained poultry slaughter protocol. At the end of each activity, the researchers discussed improvements needed to clarify the safe contained poultry slaughter procedure for low literacy audience and notes were taken. The first field trial was recorded as a written report and in the case of three field trails with women's focus groups, notes of proposed changes were made directly on a copy of the poster and as a list of suggested changes to improve understanding at the village level. At the end of each trial, the collected data were then analysed by the research team in a two‐step process: (1) would the changes in the safe contained poultry slaughter procedure improve village user understanding and be sufficiently simple to increase chances of implementation of a revised procedure at the village level, and (2) could the changes be translated into an improved outreach tool poster illustrations?

#### 2.1.1 Discovery, testing and confirmation of household implements in early safe contained poultry slaughter procedure

For the first safe contained poultry slaughter field trial, held at RLQP in Giza, the chicken was contained during the kill step in three ways—plastic bag(s), plastic bag(s) plus bucket and bucket alone. Initially, plastic bags were thought to be ideal as they are rubbish everywhere, available at no cost, and the bucket was a reasonable alternative being low cost and commonly available. For initial discovery and testing at the first meeting, plastic bags were provided to two village women for the bagged slaughter procedure development. This was conducted in an open space across the alley behind the RLQP laboratory building. A hole was poked with a finger in the bottom corner of the bag, the bag was slid over the chicken with head protruding out the hole, the bag handles were tied together to close the bag, the cervical blood vessels were severed, and the head/neck retracted down into the bag. The bag was dropped to the ground or placed in a bucket until the bird completed the involuntary muscle contractions and bleed‐out, then removed from the bag and processed. The alley was a shortcut for many pedestrians, some of whom became curious about what was happening, and they soon began to participate with ideas for how the procedure could be improved: spontaneous informal ‘crowd‐sourced’ input of ideas.

#### 2.1.2 Ensure accuracy and acceptability of safe contained poultry slaughter procedure from initial field activity at a El Fayoum governorate village

The purpose of the second safe contained poultry slaughter field trial was to determine if the conclusions from the exploratory first trial were valid, and to initiate use of a village women's focus group. It was critical that the women be fully engaged in discussion and assured that their input would be taken seriously, respected and utilized. The trial was held in an El Fayoum governorate village that had experienced HPAI zoonotic infections and deaths. The meeting had three primary phases: traditional halal slaughter to establish baseline; examination of different container types; and slaughter of chickens and ducks, as ducks are also common poultry in household flocks. All activities would be accompanied with discussion, reasoning and clarification of ‘why’ questions.

#### 2.1.3 Analyse the clarity and effectiveness of the outreach tool in field women's focus group activities in Sharkia governorate villages

**FIGURE 1:**
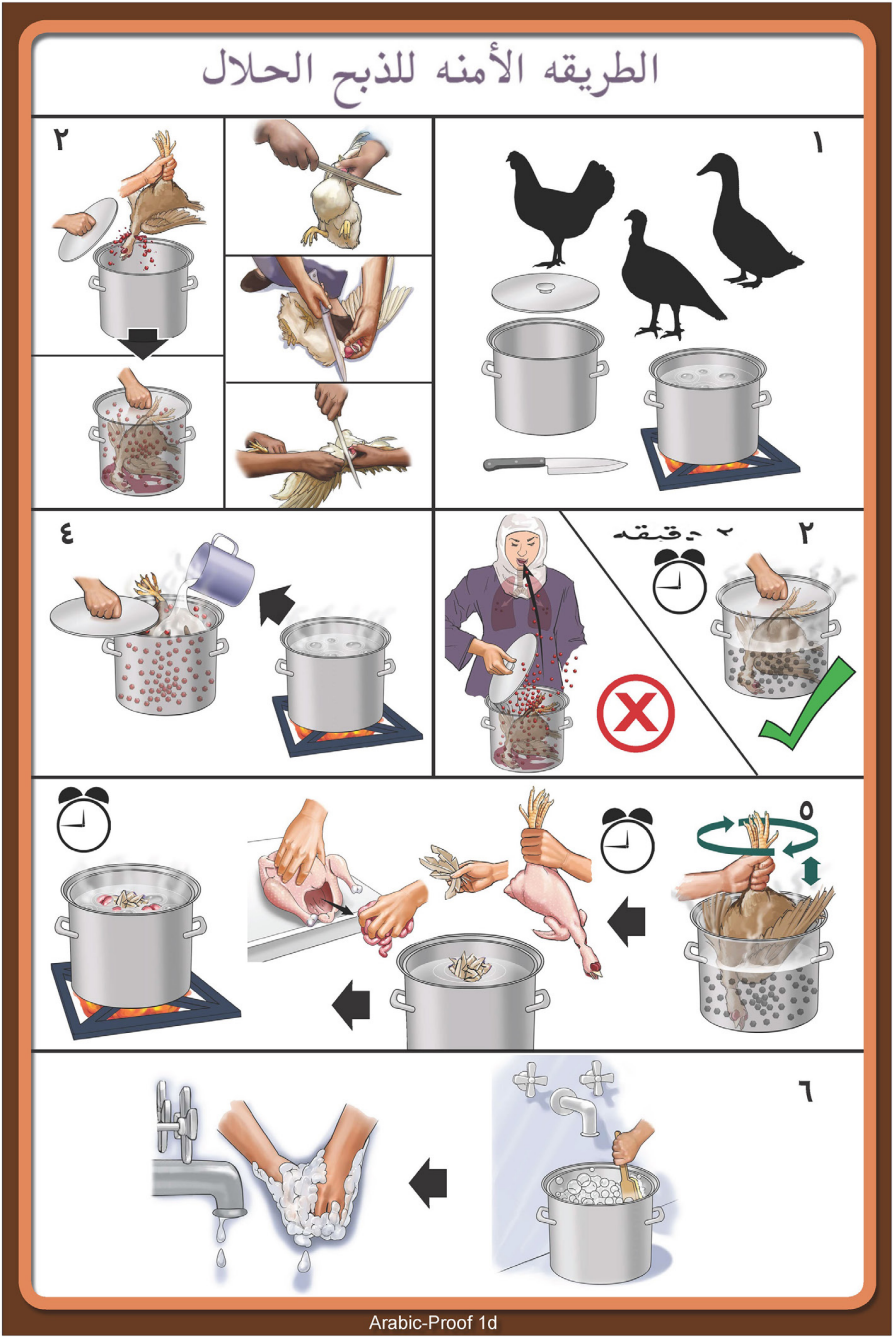
Initial Arabic poster for the Women's Focus Group in Sharkia area used for critique and improvement of the Behaviour Change Outreach Tool

**FIGURE 2:**
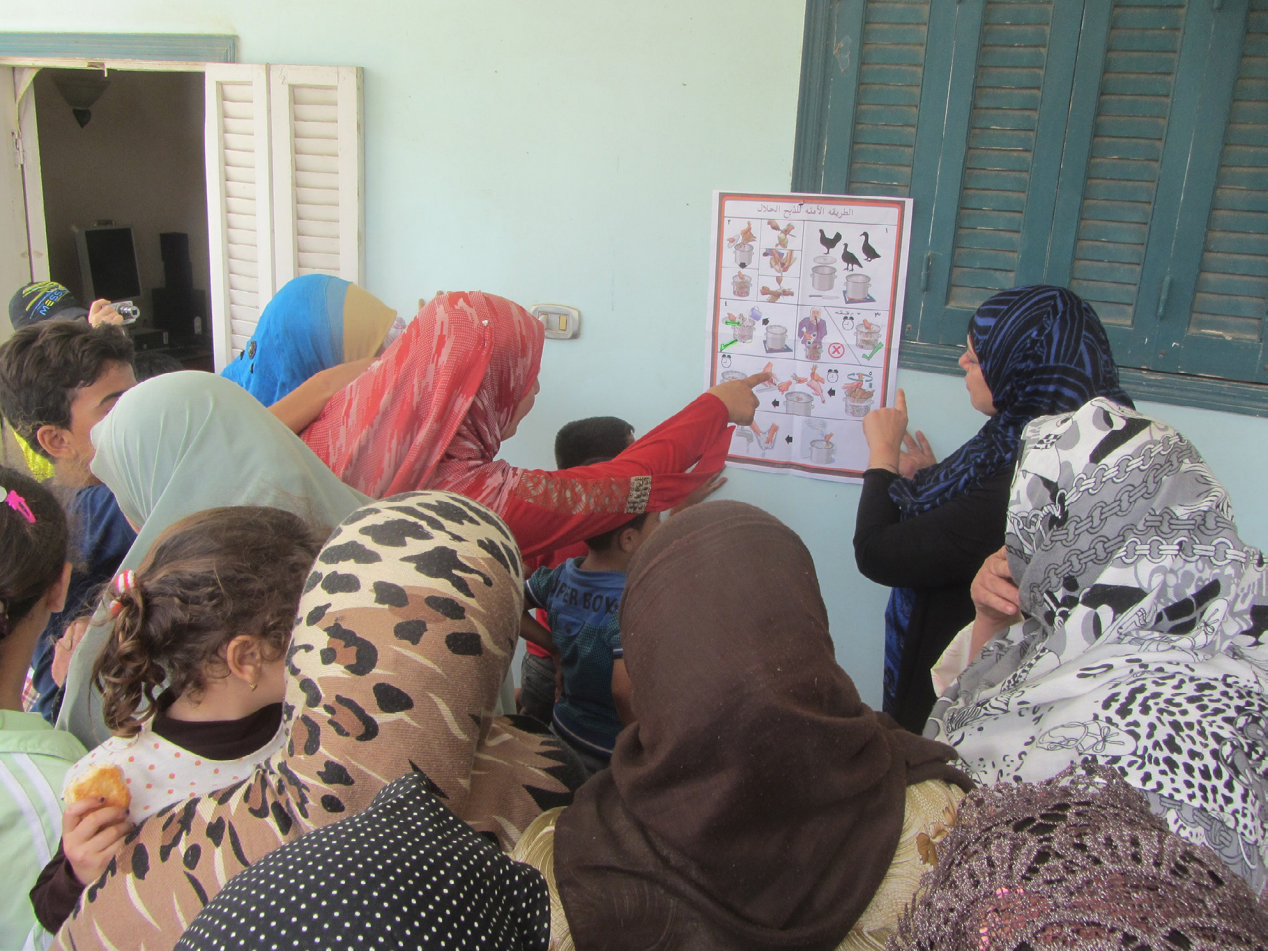
Focus and involvement of participants in Women's Focus Group in Sharkia area village concerning interpretation of illustration

The third through fifth field trials involved three target audience women's focus groups in Sharkia governorate villages which would critique the container‐based poster outreach tool (Figure 1) and fine tune the safe contained poultry slaughter procedure. Village women are often conservative in both cultural and religious matters, and the procedure was developed to be user‐friendly for them. Three villages were visited on separate days each with a group of eight women. After introductions, analysis of the poster was done with each woman individually, followed by slaughter of chickens to confirm that the procedure was well understood. For unbiased analysis, the poster was mounted on a wall without explanation to eliminate prior understanding of the message. Each woman came forward to explain what the poster meant to her (Figure 2). Throughout the process, facilitators collected information and made notes on the poster to relay back to the study designers and illustrator.

### 2.2 Behaviour change outreach tool

Outreach tool considerations revolved around practical concerns. The tool would be carried by instructors to a variety of different locations and situations, so must be easily portable, useable without electricity (thus eliminating electronic devices) and useable by instructors in front of an audience. For expanded outreach, it must be usable in a variety of venues without instructors. For multiplier effect, it must be useable as a ‘take‐it‐home’, fully understandable handout flyer. An illustrated document was developed that could be distributed in different sizes and for several purposes including as a large‐size poster for instructors; a medium‐size poster for hanging in windows of agricultural businesses, medical clinics, veterinary facilities, etc; and a small‐size handout for meeting attendees. Additionally, school children could downstream amplify the program by taking the handouts home to their mothers and sharing within the community.

Designing the storyboard was a dynamic process with first input from animal health professionals (i.e. veterinarians, laboratorians, epidemiologists and researchers) followed by discovery and testing in field trials and final critique by the women's fFocus group end‐users.

Conducting the five field trials of method development, testing and modification; and educational tool development and revision spanned an 8‐year time period (2007–2015).

## 3 RESULTS

### 3.1 Discovery, testing and confirmation of household implements in early safe contained poultry slaughter procedure

Initially, this trial incorporated bags alone, then bags and a rigid container (a bucket), and finally a bucket alone. This resulted in four outcomes:

1. Involuntary postmortem muscle contractions resulted in the claws ripping the bag, and spilling out blood, faeces and feathers. Plastic bags alone were not suitable for complete containment.
2. A mitigation to prevent claws from tearing the plastic bag would be to tether both the feet and wings. However, such restrictions are culturally and religiously not acceptable because leg and wing movements are required to ensure complete bleed‐out.
3. A hard container (e.g. bucket) in addition to the plastic bag provided consistent containment as bagging alone was unsatisfactory for containment. This led to use of a hard container alone, as bags were redundant and increased complexity.
4. Bystander critique and input about the process provided useful perspective of potential users. This crowd‐sourced information was unexpected and indicated a high level of interest that subsequent village field trials leveraged upon.

### 3.2 Ensure accuracy and acceptability of safe contained poultry slaughter procedure from initial field activity at a El Fayoum governorate village

The hostess of the meeting provided normal household implements for this exercise and the halla was introduced as a containment vessel. A halla is a multi‐purpose large aluminium pot available in various sizes ranging from 30 to 50 cm in diameter (wide base for stability) and ~20–30 cm deep. When the chicken or duck was slaughtered, dropped into the halla and the halla immediately covered, the viral plume was captured. The best cover was a commercial lid, but any rigid, flat material (e.g. flat metal pans which are common household equipment) that would completely cover the top of the halla could be used. The halla was clearly superior to plastic bags in function, universally available and entirely sufficient for viral plume capture. The plastic bag as a containment vessel was abandoned.

As women's focus groups progressed, individual steps of the safe contained poultry slaughter procedure were clarified, and the outreach tool design became more defined. When the involuntary muscle contractions were complete, for introduction of the scald water the covering lid was *slid sideways*, *never lifted*, as lifting would pull virus‐laden particles up into the face of the person and result in inhalation exposure. The protocol thereby became to slide the lid open enough to gently introduce scald water and then immediately slide the lid shut. The function of the scald water was 3‐fold: (1) loosen feathers; (2) allow steam to rise, mix with airborne virus‐laden blood droplets and aerosols and precipitate virus down into the hot water; and (3) inactivate virus. After several minutes the lid was removed, the bird grasped by the legs and stirred around the inside of the halla to saturate and loosen feathers, ensure distribution of the hot water to inactivate virus, and clean blood from the sides and bottom of the halla. The carcass was then ready for processing.

Outcomes from the first women's focus groups:

1. The halla pot, which satisfies all requirements for capture of the viral plume and village level behaviour change, was discovered. It became the standard containment vessel for slaughter of both chickens and ducks.
2. This field trial introduced women's focus group target audience participation. The women and the researchers all came to the meeting with enquiring minds and open discussion was encouraged. A free flow of ideas and creative solutions was enhanced and suggestions for the outreach tool were generated. There was a comfortable ambience between researchers and women's focus group participants that created confidence, honest appraisal and enthusiastic participation.
3. Prior to this field activity, one researcher not previously involved suggested that acceptance of the safe contained poultry slaughter procedure would be low, as it would change traditions that had existed for centuries. However, at the end of the day when the women were asked ‘will you use this procedure?’ the answer was a resounding ‘Of course we will do this! We have seen our friends die from this disease!’
4. After completing the work, a complementary lunch featuring fresh fried chicken and duck was provided by the hostess for everyone involved as celebration of their participation.

Based on what was learned in this second field trial and with advice of animal health professionals, an educational multi‐panel poster was developed (Figure 1) using a rigid containment vessel for slaughter confinement. All further illustrations used the halla as the standard containment vessel.

### 3.3 Analyse the clarity and effectiveness of the outreach tool in field women's focus group activities in Sharkia governorate villages

In the three women's focus groups in the Sharkia area, what the researchers thought a near‐perfect set of illustrations was critiqued by the women and 35 changes were proposed (Figure 2). Some suggestions were minor, but nonetheless important. The level of detail indicated a great deal of attentiveness by the women. (Table 1). Throughout these meetings, there was discussion, information exchange and critique. The women were engaged and articulate in helping the researchers with analysis.

**TABLE 1:** Outcomes from women's focus groups in Sharkia area villages to improve clarity of Figure 2 poster in pictoral education tool to the final posters in Arabic and English versions

Points of improvement for Figure 1 from women's focus groups in Sharkia Area	Changes in Final 11‐Panel Arabic and English Versions
Separate all panels with a space	Used in both versions
2 Use arrows to indicate the flow which should be right‐to‐left, down, left‐to‐right, down, etc.; that is flow like a ‘snake’	Arabic, right‐to‐left, and English, left‐to‐right; both ‘snake’ flow
3 Number the pages in Arabic, upper right corner	Used in both versions
4 Reduce size of Panel 1	Eliminated panel in both
5 In Panel 1 use pictures or illustrations of the birds and not silhouettes	Eliminated panel in both
6 Use halla‐shaped pot for bird processing and a different shaped pot to heat the water. Limit the hot water pot presence to only when hot water is used	Used in both versions
7 In all illustrations of pots with water, make the water blue.	Used in both versions
8 In illustrations with boiling water, make more bubbles and more steam for clarity	Used in both versions
9 Make boiling pot transparent to show viscera and feathers inside	Used in both versions
10 Make feathers bigger to assure their identity	Used in both versions
11 Add more detail on feathers	Used in both versions
12 Because transparent lids are confusing, make all lids solid	Used in both versions
13 Make all blood a very bright red	Used in both versions
14 In the illustration of woman inhaling virus add more virus particles and as bright red monsters	Between panel # 3 and 4–both versions
15 Make the virus into nasty little monsters like the bacteria in toothpaste advertisements—red for living and black for dead	Not used as small size of virus particles prohibited details of ‘monster’ viruses
16 In the illustration of woman inhaling virus add two red Xs on lid	Modified on Arabic version to one large red X on panel
17 Use barbed arrows, one going each way, to illustrate the back‐and‐forth sliding motion of the lid	Panel #4—both versions
18 In ladling water for scald step, make pots on equal size and use long arrow from boiling water through ladle and into pot to show the continuous motion	Panel #4—both versions
19 Illustration about scald to loosen feathers—add a hand with a wooden spoon pushing the bird down into the scald water	Not used as wooden spoon would contradict the critical closed lid concept for containing the virus
20 Enlarge the clock in all panels	Panel #5–both versions
21 Remove red wattles in illustrations where the virus is black—it confused the dead virus issue	Panels #5/6–changed wattle colour to pale pink
22 Defeathering is done in a pan and the carcass is then rinsed in another pan, eviscerated, edible organs sorted away, intestines put with the feathers, and the clean carcass with edible organs kept in the rinse pan	Panel #7—sequence redone
23 Leave some feathers on the carcass in the defeathering panel	Panel #7—both versions
24 Evisceration panel include removal and saving of edible organs—gizzard, heart and liver with upward rotation of hand holding viscera to ensure clarity	Panel #7–edible viscera added with flow arrow in both versions
25 Rotate hand with intestinal viscera upwards to clarify removal	Panel #7—Additional flow arrow added for clarification
26 Add more viscera and feathers in the pot to be boiled	Panel #7—both versions
27 Boiling viscera and feathers to show destruction of virus	Panel #8—both versions
28 Include a panel with the finished product—the chicken carcass and the edible organs	Panel #9—both versions
29 10—Make the clean‐up a single separate panel	Panel #10—both versions
30 10—Add detergent container, have bubbles falling over side, add a cleaning pad, and eliminate the wooded handled brush in pot section of Clean‐up panel	Panel #10—both versions
31 10—Less foam on hands and add a bar of soap for handwashing in Clean‐up panel	Panel #10—both versions
32 Include the knife in the clean‐up panel	Unintended omission but deemed not essential
33 Develop a 5‐panel poster listing the four critical to‐do and one do‐not‐do actions, and in the 11‐Panel poster, label the actions with a green circle around a green check mark and red circle around a red X, respectively	Used in both versions
34 For the five (5) critical actions have them as separate panels on the poster with green borders for the ‘what to do’ and red edges for the ‘what to NOT do’. Those are as follows: 1) Contain the virus (green); 2) Do not lift the lid (red); 3) Slide the lid (green); 4) Inactivate the virus in the container (green); 5) Inactivate the virus in the waste feathers and viscera;	Used in both versions
35 Print the 5‐panel poster on the back of 11‐panel poster or as a separate handout	Used in both versions

One suggestion was especially important and a revelation to the designers. The poster had four horizontal lines each with multiple panels. As in Arabic writing, the first illustration was at the top right and the progression was right‐to‐left, down, right‐to‐left, down. One woman began at the top right, went across the three panels of the first line, was then lost and confused. Why? When explained that the poster went right‐to‐left, down, etc., like reading, she objected to the design. ‘Why does it jerk back and forth like that? Life is a flow. It should go along like life. The flow should be right‐to‐left, down, left‐to‐right, down, right‐to‐left, down, left‐to‐right ‐ so that it flows along like life, or like a snake’. This observation by a low‐literate participant was the most significant and important of the 35 recommendations for improvement, causing a complete re‐design of the poster.

**FIGURE 3:**
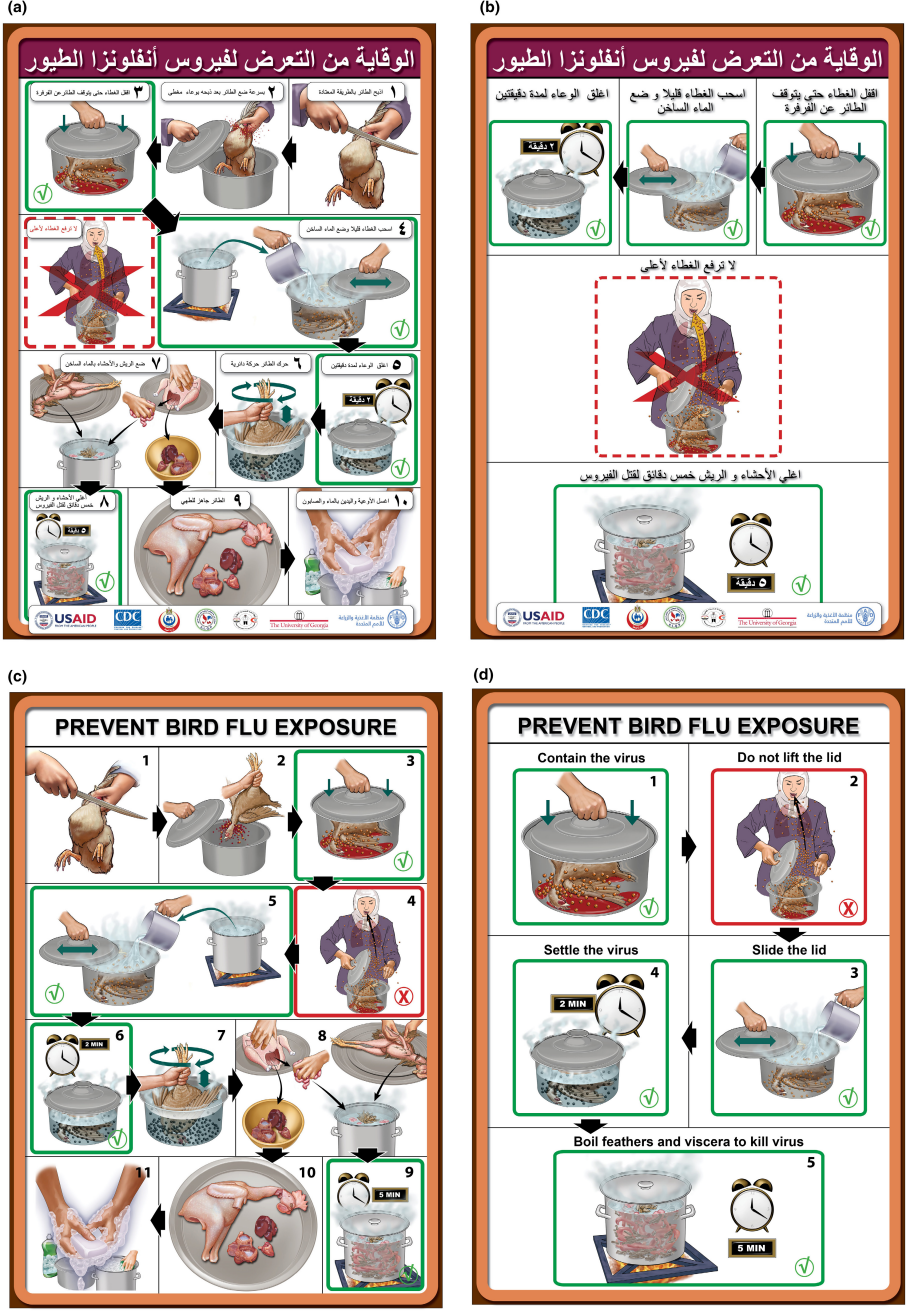
Final 11 and 5 panel posters for the safe contained poultry slaughter procedure in Arabic (a and b) for use in Egypt and English (c and d) version, useable for adaptation to other languages, cultures, and religious beliefs

The outcome of these three women's focus groups demonstrated the importance of target audience advice. The suggested changes were incorporated into the poster and led to the final Arabic poster design (Figure 3a,b) and an English version for global distribution of the educational concepts (Figure 3c,d).

### 3.4 Behaviour change outreach tool

The first iteration of the poster was a 6‐panel cut‐and‐paste black and white storyboard of the bagged slaughter procedure that was never used because rigid containers became the implement of choice.

Beginning with second field trial, the multi‐step poster visually described a specific ‘Education Principle’ for each step of safe contained poultry slaughter procedure to ensure complete understanding of the entire procedure. This curriculum organized progression of the steps and enhanced sequencing for the posters with use of the halla.

The three field trials in Sharkia were target audience women's focus group events. The poster was shown to the women for their critique to determine whether the illustrations created understanding (Figure 2). Immediate notes were recorded on the poster (Table 1). A gratuity of a chicken and/or duck and a new bucket was given to each participant.

The storyboard began with a simple draft storyboard and was revised through 33 iterations. The end‐product was an 11‐panel poster of the safe contained poultry slaughter procedure and a 5‐panel summary poster of the five most important steps. Posters were produced in Arabic (Figure 3a,b) and English (Figure 3c,d). The 11 and 5 panel posters were designed to be printed front and back.

Total costs for each meeting were minimal, around $150. Expenses included vehicular transport to the villages, HPAI virus test‐negative chickens and ducks for procedure demonstration, and a gratuity bucket for each participant in the women's focus groups. Equipment used for safe contained poultry slaughter procedure was all local household utensils brought by the women.

The Islamic Authority in Egypt was requested to review the procedure for religious acceptance within the current halal slaughter framework. The Secretariat of the Egyptian Fatwa House legitimized the procedure as acceptable halal slaughter, that is the safe contained poultry slaughter procedure was found to be in religious compliance and people were instructed to use the procedure.

## 4 DISCUSSION

In response to the high number of human H5N1 influenza cases in women and children in Egypt, this study developed a halal slaughter compliant procedure for reducing risk of airborne zoonotic HPAIV infections transmitted during home poultry slaughter. The basic concept questions asked included ‘can home slaughter of asymptomatic HPAIV‐infected poultry generate airborne virus and cause transmission to humans, and can this risk be reduced by simple changes in procedures utilizing common household implements?’ In support and parallel with the current study, airborne virus was demonstrated during simulated home slaughter in a high biosecurity laboratory and potential mitigations were examined and verified as to their ability to reduce airborne virus generation and zoonotic transmission in a ferret model (Bertran et al., 2017; Bertran et al., 2018). In translating the laboratory outcomes, the current safe contained poultry slaughter procedure was confirmed to be effective, affordable, practical and sustainable. The focus for sustainability necessitated the use of equipment commonly existing in Egyptian households and with minimal modification of existing slaughter protocols. This safe contained poultry slaughter procedure was translated into a simple pictorial education tool intended for a limited literacy village audience.

The safe contained poultry slaughter procedure and behaviour change educational tool were developed concurrently through three phases: (1) discovery, testing and confirmation of household implements for airborne virus capture during the kill and scald steps and design of first draft storyboards for evaluation; (2) field implementation for determining accuracy and acceptability of safe slaughter procedure and use of the illustrated poster as the educational tool of choice; and (3) analyse clarity and effectiveness of the outreach tool with village level women's focus groups and final modifications. Initially, the pictorial educational tool was designed by college‐educated veterinarians, laboratorians, and epidemiologists. The educational tool was developed based on scientific principles. However, for cultural acceptance, the materials were modified to an easy‐to‐understand format comprehended by both low literacy and literate audiences, ranging from elementary age children to adults in Egypt. The critical final step was the input of women's focus groups, including their children, who refocused the process on simplicity and understandability, and prompted changes in content and structure of pictorial panels within the storyboards to convey an accurate message.

The educational tool was developed for the low literacy rural Egyptian audience with limitations to its immediate practical use both culturally and religiously in other geographic locations. For use in other cultures, the outreach communication tools were designed to be easily changed to fit any society in the world in terms of clothing, skin tones, facial characteristics and traditional equipment. Accordingly, wherever a poultry‐borne zoonotic disease associated with human inhalant exposure might occur, the outreach tools can be transformed and made specific to that region and those people in order to see themselves in the illustrations and feel comfortable with the lifesaving message.

Since completion of the safe contained poultry slaughter procedure and the educational tool, the 11 and 5 panel Arabic and English posters has been provided to multiple non‐governmental organizations for potential use. For example, in November 2015 the United Nations Food and Agriculture Organization (FAO) in Egypt utilized the posters in their Exposure Reduction Program (FAO, 2016c). A field force of 823 veterinary staff performed 6701 educational seminars and distributed over 1.44 million A4 size handout flyers at meetings and as a ‘take one’ in agricultural supply stores and other venues. In addition, 9700 sticky‐back posters were printed for display throughout Egypt. Between these modes of outreach, direct contact at meetings and the trickle‐down woman‐to‐woman conversations using the handouts, an estimated seven million persons were reached with the education program. This Exposure Reduction Program was associated with a decline in human cases from 136 cases in 2015 to 10 in 2016, 3 in 2017 and zero in 2018, 2019 and 2020 (WHO, 2022). However, beginning in late 2017 and onwards, a replacement of the zoonotic‐potential H5N1 HPAIV in Egypt with a less zoonotic strain may have contributed to the maintenance of reduced human cases as compared to 2015. It is unclear if other factors contributed to the decline in zoonotic cases in 2016 and in more recent years.

This study created understanding and a simple solution for a non‐scientific community about a life‐threatening disease. From the originating idea to development of the contained poultry slaughter procedure and educational tool, the study was conceived, designed and implemented by veterinarians for protection of humans in a One Health framework. The study engaged women, the target audience, specifically for safety and protection of themselves and their families from HPAI, and for their continuing important contributions to family nutrition and income via their household poultry flocks. Use of this simple education tool with adaptation to local cultural and religious conditions can have a broader impact on prevention of HPAI zoonotic infections in affected village or rural communities around the world.

## FUNDING INFORMATION

United States Department of Agriculture/Animal and Plant Health Inspection Service/International Services (USDA/APHIS/IS); Egyptian Reference Laboratory for Quality Control of Poultry Production (RLQP), Animal Health Research Institute (AHRI), Agricultural Research Centre (ARC); USDA/Agricultural Research Service (ARS) Southeast Poultry Research Laboratory (SEPRL); United States Agency for International Development (USAID); Funding was provided by USDA/ARS project #6040‐32000‐048‐00D and #6040‐32000‐063‐00D and by USDA/APHIS/International Services (Clark contract salary). The funders had no role in the design of the study; in the collection, analyses or interpretation of data; in the writing of the manuscript, or in the decision to publish the results. All authors have read and approved the contents of the submitted manuscript.

## CONFLICT OF INTEREST

The authors declare they have no conflict of interest.

## ETHICAL APPROVAL

The field poultry studies and women's focus groups had ethical approval through the Animal Health Research Institute, Ministry of Agriculture in Egypt.

## Data Availability

The data that support the findings of this study are available from the corresponding authors upon reasonable request.
